# Protective effect of melatonin against blue light‐induced cell damage via the TRPV1–YAP pathway in cultured human epidermal keratinocytes

**DOI:** 10.1002/biof.70015

**Published:** 2025-04-04

**Authors:** Seoyoung Choi, Eunbi Yu, See‐Hyoung Park, Sae Woong Oh, Kitae Kwon, Gyeonghyeon Kim, Heejun Ha, Hee Seon Shin, Seokhyeon Min, Minkyung Song, Jae Youl Cho, Jongsung Lee

**Affiliations:** ^1^ Molecular Dermatology Laboratory, Department of Integrative Biotechnology, College of Biotechnology and Bioengineering Sungkyunkwan University Suwon City Korea; ^2^ Department of Bio and Chemical Engineering Hongik University Sejong City Korea; ^3^ Integrative Research of T cells Laboratory, Department of Integrative Biotechnology, College of Biotechnology and Bioengineering Sungkyunkwan University Suwon City Korea; ^4^ Department of Biopharmaceutical Convergence Sungkyunkwan University Suwon City Korea; ^5^ Molecular Immunology Laboratory, Department of Integrative Biotechnology, College of Biotechnology and Bioengineering Sungkyunkwan University Suwon City Korea

**Keywords:** blue light, mammalian sterile 20‐like kinase 1/2 (MST1/2), melatonin, transient receptor potential vanilloid 1 (TRPV1), yes‐associated protein (YAP)

## Abstract

Although blue light has been known to negatively affect skin cells, its detailed signaling mechanisms and anti‐blue light agents have not been clearly elucidated. We investigated the involvement of Yes‐associated protein (YAP)‐mediated Hippo signaling in blue light‐induced apoptosis, depending on the degree of blue light exposure. Additionally, we elucidated the effects of melatonin on blue light‐irradiated keratinocytes and examined their action mechanisms. After blue light irradiation, its effects and antagonizing effects of melatonin on cell proliferation, apoptosis, DNA damage, and transient receptor potential vanilloid 1 (TRPV1)/YAP‐mediated signaling were examined in HaCaT cells using western blots, image analysis, flow cytometric analysis, co‐immunoprecipitation, and immunocytochemistry. We found that melatonin treatment attenuated the reduced cell viability and increased production of reactive oxygen species (ROS) in response to blue light irradiation. In the experiments to investigate the mechanism of action of blue light and melatonin, we found that YAP changed its binding protein, either p73 or TEAD, depending on the degree of blue light exposure. Melatonin treatment reduced blue light‐induced phosphorylation of TRPV1 and MST1/2. Upon treatment with capsazepine, an antagonist of TRPV1, MST1/2 activation also reduced. Furthermore, we found that prolonged blue light irradiation induced DNA damage, which in turn induced YAP–p73 nuclear translocation. These effects were also notably attenuated by melatonin. These findings indicate that depending on the duration of blue light irradiation, two different YAP‐mediated Hippo signaling pathways are activated. Additionally, these findings suggest that melatonin could be a potential therapeutic agent for blue light‐induced skin damage.

## Graphical abstract

Melatonin protects human keratinocytes from blue light‐induced damage by inhibiting TRPV1‐mediated ROS production and modulating YAP‐dependent Hippo signaling. Depending on exposure duration, YAP interacts with TEAD to promote proliferation or with p73 to induce apoptosis. Melatonin mitigates these effects, reducing DNA damage and cell death.

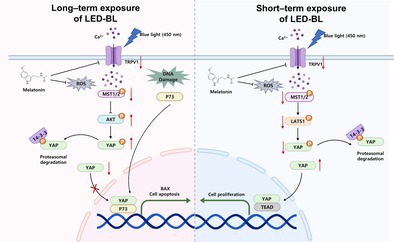

**Abbreviations**
ABTS: 2,2′‐azino‐bis(3‐ethylbenzothiazoline‐6‐sulfonic acid)
Ca^2+^
: calcium ion
DCF‐DA: A 2′,7′‐dichlorofluorescein diacetate
DMEM: Dulbecco's modified Eagle's medium
GAPDH: glyceraldehyde 3‐phosphate dehydrogenase
HaCaT: human keratinocytes
LED‐BL: blue light‐emitting diode
MST1/2: mammalian sterile 20‐like kinase 1/2
PBS: phosphate‐buffered saline
ROS: reactive oxygen species
TEAD: TEA domain transcription factor
TRPV1: transient receptor potential cation channel subfamily V member 1
UV: ultraviolet
YAP: Yes‐associated protein

## 1 INTRODUCTION

Skin is an important organ that protects our body against the external environment; therefore, it constantly interacts with extrinsic damaging factors, such as pathogens, chemicals, and radiation.^1^, ^2^ These harmful stresses induce inflammatory responses and oxidative stress, leading to the disruption of skin homeostasis.^3^ Hence, characterizing the signal transduction induced by external stresses and developing effective treatments is a significant issue in skin research.

Blue light within the spectrum of visible light has the shortest wavelength (400–500 nm) and highest energy. Recently, it has been reported that 60% of people spend over 6 h a day in front of digital devices, which emit blue light that negatively impacts retinal cells.^4^ Several studies have examined how ultraviolet (UV) radiation affects skin cells. UV light occupies only 3% of the total solar energy spectrum, whereas visible light occupies a greater proportion at 42%.^5^ In addition, blue light can penetrate deeper into the dermis than UV light and affect skin physiology by inducing DNA damage and oxidative stress.^4^, ^6^

Transient receptor potential vanilloid 1 (TRPV1), a member of the transient receptor potential cation channel (TRP) family, is a non‐selective cation receptor that reacts with capsaicin. It is expressed in keratinocytes, kidney cells, primary sensory neurons, and bronchial epithelial cells.^7^, ^8^ TRPV1 is also activated by several factors such as UV light, blue light, low pH, and high temperatures (>42°C).^8^, ^9^ Activated TRPV1 induces calcium ion influx and mediates multiple signaling pathways. Generally, it prevents cell proliferation, promotes differentiation, and induces the production of several proinflammatory cytokines.^8^, ^10^, ^11^ Additionally, in our previous study, we showed that upregulation of TRPV1, a blocker of EGFR‐FoxO3a signaling, mediates the effects of blue light on keratinocyte proliferation. Notably, the increased calcium influx resulting from TRPV1 activation is another mechanism by which blue light induces the production of reactive oxygen species (ROS).^9^

MST1 and MST2 (MST1/2) are important components of the Hippo signaling cascade, which plays a critical role in the self‐renewal of stem cells, regeneration of tissues, and control of organ size.^12^ MST1/2 can be activated through ROS and calcium influx, leading to the inhibition of cell proliferation.^13^, ^14^ A kinase cascade activated by MST1/2 kinases and nuclear Dbf2‐related family kinases, LATS1 and LATS2 (LATS1/2) proteins, also plays a central role in the Hippo signaling cascade. MST1/2 subsequently phosphorylate and activate LATS1/2, which then phosphorylate Yes‐associated protein (YAP) in the serine 127 region.^12^ Phosphorylated YAP is either degraded or sequestered by proteases and 14‐3‐3 proteins in the cytoplasm.^15^, ^16^

YAP is a downstream molecule in Hippo signaling and a coactivator that collaborates with several transcription factors to promote gene transcription, specifically those involved in the regulation of cell proliferation.^17^, ^18^ For instance, YAP forms a functional hybrid transcription factor with the TEA domain transcription factor (TEAD) to induce the expression of pro‐survival and pro‐proliferative genes, leading to cell proliferation.^19^ Conversely, in response to DNA damage, YAP can form a complex with the p73 transcription factor and induce cell apoptosis.^20^, ^21^ Following this, Akt phosphorylates YAP at serine 127, sequestering YAP in the cytoplasm and blocking its interaction with p73.^22^ Akt activation can be inhibited by MST1/2 activation, thus controlling the extent of cell proliferation.^22^ The contradictory roles of YAP as both a tumor promoter and tumor suppressor appear to vary depending on the type of cell and signaling.

Melatonin (N‐acetyl‐5‐methoxy tryptamine) is an ancient and highly conserved antioxidant that has remained unchanged throughout evolution, existing in all species from cyanobacteria to humans. In mammals, it rapidly disappears from circulation, accumulating in mitochondria, where its concentration is significantly higher than in the blood. Its high concentration and multifunctional antioxidant properties protect mitochondria from oxidative stress and free radical damage.^23^ It is also a well‐known antioxidant molecule produced and metabolized in the skin.^24^, ^25^ Melatonin improves UV‐induced oxidative damage and inflammatory conditions by blocking nuclear factor (NF)‐κB, interleukin (IL)‐6, and other inflammatory cytokines.^26^, ^27^

The skin acts as a peripheral neuroendocrine organ, producing various hormones, neurotransmitters, and peptides, including melatonin to regulate responses to environmental factors which relate the action of melatonin with UVR. These mediators, along with their receptors, interact through local and central pathways like the HPA axis to maintain homeostasis and adapt to UV‐induced stress.^28^

Recent studies have reported that blue light suppresses the proliferation of skin keratinocytes and increases ROS production by activating TRPV1.^9^ Given that blue light causes skin damage, close attention has been paid to identifying a molecule that can inhibit blue light signaling. Therefore, this study was aimed to examine the effects of melatonin on blue light‐induced cell damage. In particular, we examined novel signaling pathways of blue light‐induced effects and the inhibitory effects of melatonin on TRPV1 and its downstream effectors.

## 2 MATERIALS AND METHODS

### 2.1 Cell culture and materials

HaCaT cells (American Type Culture Collection, Manassas, VA, USA), a human keratinocyte cell line, were cultured in Dulbecco's modified Eagle's medium (DMEM) supplemented with 10% fetal bovine serum and 1% antibiotics (penicillin/streptomycin). These cultured cells were maintained in a humidified 5% CO_2_ incubator at 37°C.

The following antibodies were used for western blot analysis. Antibodies against TRPV1 (1:1000 dilution, PA1‐29421) and p‐TRPV1 (Ser502) (1:1000 dilution, PA5‐64860) were obtained from Invitrogen (Carlsbad, CA, USA). Antibodies against p‐MST 1 (Thr183)/MST 2 (Thr180) (1:1000 dilution, bs‐3294R) were purchased from Bioss Inc. (Wo‐burn, MA, USA). p‐LATS1 (1:1000 dilution, #8654), p‐YAP (Ser127) (1:1000 dilution, #4911), YAP (1:1000 dilution, #14074), p73 (1:1000 dilution, #14620), TEAD (1:1000 dilution, #13295), phospho‐histone H2AX (Ser139) (1:1000 dilution, #9718), AKT (1:1000 dilution, #9272), and p‐AKT (Ser473) (1:1000 dilution, #12694) were purchased from Cell Signaling Technology (CST, Danvers, MA, USA). Bax (1:1000 dilution; sc‐7480) was purchased from Santa Cruz Biotechnology (Dallas, TX, USA). α‐Tubulin (1:10,000 dilution, ab7291) was obtained from Abcam (Cambridge, UK). Antibodies against β‐actin (1:4000 dilution, A5316), anti‐rabbit immunoglobulin G (IgG) (1:4000 dilution, A0545), and anti‐mouse IgG (1:4000 dilution, A9044) were purchased from Sigma–Aldrich (St. Louis, MO, USA).

### 2.2 Blue light irradiation and melatonin treatment

Melatonin was obtained from Sigma–Aldrich (M5250, St. Louis, MO, USA). The stock of melatonin was prepared in 100% EtOH (64‐17‐5, Sigma–Aldrich, St. Louis, MO, USA) at concentrations of 10, 100, and 200 mM, which were further diluted to 10, 100, and 200 μM in DMEM for cell application.

The cells were exposed to a blue light‐emitting diode (LED‐BL) having an emission peak of 470–480 nm and a power density of 76 W/m^2^ at 20°C by using a photoreactor (CCP‐4V, Luzchem, Ottawa, ON, Canada).

Long‐term exposure to blue light with melatonin treatment:

1st day: The cells were pretreated with melatonin (10, 100, and 200 μM) or the control (0.1% EtOH) for 24 h in a phenol red‐free culture medium (PRFCM) before blue light irradiation.

2nd day: After washing the cells with phosphate‐buffered saline (PBS), media renewal, and blue light irradiation for 30 min (13.68 J/cm^2^) were conducted. After irradiation, the cells were incubated with melatonin (10, 100, and 200 μM) for 24 h.

3rd day: The media were changed to phenol red‐free DMEM, and the cells were subsequently irradiated for 30 min with blue light. After irradiation, the cells were immediately treated with melatonin (10, 100, and 200 μM) and incubated for 24 h.

Short‐term exposure to blue light with melatonin treatment:

The cells were washed with PBS, and PRFCM renewal and blue light irradiation for 30 min were conducted. After blue light irradiation, melatonin stock (1000×) was added immediately and incubated for 60 min.

### 2.3 Cell titer Glo® 2.0 assay for cell viability analysis

The cells were plated in an opaque‐walled 96‐well plate with PRFCM. Then, they were treated with melatonin for 24 h; subsequently, the cells were irradiated with blue light (13.68 J/cm^2^) followed by another treatment with melatonin incubated for 24 h, which was repeated for 2 days. After all treatments, the cells were incubated for 24 h in a 5% CO_2_ incubator at 37°C. After incubation, 100 μL of Cell Titer Glo® 2.0 reagent (G9242, Promega, Madison, WI, USA) was added to each well (with each containing an equal amount of culture medium). Cell lysis was performed by shaking the contents for 2 min before incubating the cells at 20°C for 10 min. Luminescence was then measured using a microplate reader (Synergy HTX Multi‐Mode Reader, BioTek, Winooski, VT, USA).

### 2.4 Fluo‐4 Ca^2+^ influx assay

The cells were cultured to near confluence in 96‐well black‐wall/clear‐bottom microplates, followed by treatment with melatonin or capsazepine, both of which were 1000× diluted in assay buffer for 1 h. After treatment, the assay buffer was removed from cell cultures, and the cells were stained with 1× Fluo‐4 NW Ca^2+^ reagent‐loading solution (F36205, Invitrogen, Waltham, MA, USA) at 37°C for 30 min and then incubated for another 30 min at 20°C. After incubation, the cells were irradiated with blue light for 10 min (4.56 J/cm^2^) and then treated with melatonin dissolved in assay buffer, except for the control group. After treatment, fluorescence was measured at an excitation wavelength of 494 nm and an emission wavelength of 516 nm. After background subtraction, the change in fluorescence was calculated as a percentage of control fluorescence.

### 2.5 DCF‐DA cellular ROS detection assay

A 2',7'‐dichlorofluorescein diacetate (DCF‐DA) cellular ROS detection assay kit (ab113851; Abcam, Cambridge, UK) was used to quantify ROS generation following the manufacturer's instructions. The cells were seeded in 96‐well black‐wall/clear‐bottom microplates and were pretreated with melatonin (10, 100, 200 μM) or control (EtOH) in PRFCM for 24 h. After treatment, the cells were washed twice with PBS and stained with 20 μM DCF‐DA in PBS for 30 min at 20°C under light‐blocked conditions. The DCF‐DA solution was discarded after incubation, and the cells were washed with PBS. Then, PRFCM was added to each well, and the cells were irradiated with blue light for 30 min (13.68 J/cm^2^). Fluorescence was measured at an Ex/Em ratio of 494/516 nm using a microplate reader. The fluorescence signal, as an indicator of intracellular oxidants, was measured and compared to that of the respective non‐exposed control cells to characterize the level of oxidative stress and assess the changes in intracellular ROS levels induced by LED‐BL and melatonin.

### 2.6 2,2′‐Azino‐bis(3‐ethylbenzothiazoline‐6‐sulfonic acid) radical scavenging activity

Free radical scavenging activity of melatonin was determined by ABTS (2,2′‐azino‐bis (3‐ethylbenzothiazoline‐6‐sulfonic acid)) radical cation decolorization assay. ABTS + cation radical was produced by the reaction between 7 mM ABTS (30931‐67‐0, Sigma‐Aldrich, St. Louis, MO, USA) in water and 2.45 mM potassium persulfate (7727‐21‐1, Sigma‐Aldrich, St. Louis, MO, USA) (1:1), stored in the dark at 20°C for 12–16 h before use. ABTS + solution was then diluted with water to obtain an absorbance of 0.700 at 734 nm. Different concentrations of melatonin were added separately to ABTS· + solution. 150 μM Vitamin C was introduced as the positive control. After 30 min of incubation at 20°C, samples were measured at 734 nm absorbance. The radical scavenging activity was calculated using the following equation:

$$\text{ABTS scavenging activity}=\frac{\text{Absorbance of control}-\text{Absorbance of sample}}{\text{Absorbance of control}}$$

### 2.7 Nuclear and cytoplasmic fraction

Western blotting was performed to confirm the translocation of transcription factors within the nuclear and cytoplasmic fractions by using NE‐PER Nuclear and Cytoplasmic Extraction Reagents (78833; Thermo Fisher Scientific, Waltham, MA, USA), respectively, according to the manufacturer's instructions.

### 2.8 Western blotting analysis

HaCaT cells were plated on 60 π plates and treated with melatonin and blue light as described above. The cells were harvested and centrifuged at 15,928*g* for 5 min. The supernatant was discarded, and the cells were lysed in RIPA lysis buffer [25 mM Tris–HCl [pH 7.6], 150 mM NaCl, 1% NP‐40, 1% sodium deoxycholate, and 0.1% SDS] (Thermo Fisher Scientific, Waltham, MA, USA), which included Halt protease and a phosphatase inhibitor cocktail (Thermo Fisher Scientific, Waltham, MA, USA). Briefly, 6%–10% SDS electrophoresis was used to separate the recovered proteins, which were then transferred to a polyvinylidene difluoride (PVDF) membrane (162‐0177, Bio‐Rad, CA, USA). The membrane was then blocked with a 2% bovine serum albumin (BSA) solution for 1 h before being treated with primary antibodies overnight at 4°C. The membrane was washed three times with Tris‐buffered saline containing Tween 20 and probed for 1 h at 20°C with secondary antibodies. ECL western blotting reagents (170‐5061, Bio‐Rad, Hercules, CA, USA) were used to visualize the blots.

### 2.9 Immunocytochemistry

The cells were fixed for 15 min with 4% paraformaldehyde in PBS and then permeabilized for 20 min at 20°C with 0.1% Triton X‐100 and 0.01% Tween 20 in PBS. After blocking the cells with 3% BSA in PBS, the cells were incubated with Anti‐YAP (1:100 dilutions, 14074, CST, Danvers, MA, USA) antibodies for 24 h at 4°C. Subsequently, the cells were washed three times with PBS and incubated with Flamma‐594 secondary antibodies (Abcam, Cambridge, UK). Finally, after being counterstained with DAPI, the cells were mounted on glass slides and imaged using a Zeiss LSM 700 laser scanning confocal microscope with a C‐Apochromat 20× objective (Zeiss, Jena, Germany). Images were captured at the same laser power, and the mean intensity of the fluorescence signals was evaluated to determine the immunofluorescence intensity. ZEN 2012 Blue (Zeiss) and ImageJ software 1.53e (National Institutes of Health, Bethesda, MD, USA) were both used to analyze the data with the same processing parameters.

### 2.10 Co‐immunoprecipitation

HaCaT cells were grown in 100 mm dishes to 70%–80% confluency and exposed to blue light for 3 days, with or without melatonin treatment. Whole‐cell extracts were prepared in IP lysis buffer (25 mM Tris–HCl pH 7.4, 150 mM NaCl, 1 mM EDTA, 1% NP‐40, 5% glycerol, 10% protease phosphatase inhibitor, and 1 mM PMSF). Dynabeads™ magnetic beads (1003D, Thermo Fisher Scientific, Waltham, MA, USA), which had been preincubated with anti‐YAP antibody (1:100 dilutions, 14074, CST, Danvers, MA, USA) were added to whole‐cell lysates and incubated for 24 h at 4°C. Afterwards, the beads were washed in PBS (pH 7.4) containing 0.1% Tween 20 and eluted with elution buffer (50 mM glycine, pH 2.8). The immunoprecipitated samples were analyzed using western blotting with an anti‐p73 antibody. The blots were reprobed with an anti‐YAP antibody to quantify the amount of immunoprecipitated YAP protein.

### 2.11 Edu incorporation assay

Using Click‐iT™ EdU cell proliferation kit (A10044, Invitrogen, Waltham, MA, USA) for imaging, EdU incorporation assays for cell proliferation analysis were carried out in accordance with the manufacturer's instructions. After adding 10 μM of EdU to cells cultured on glass coverslips, the coverslips were incubated for 12 h. The cells were then permeabilized for 20 min at 20°C in 0.1% Triton X‐100 and 0.01% Tween 20 after being rinsed three times in PBS, and fixed for 20 min in 4% paraformaldehyde in PBS. The cells were then stained with the Click‐iT® reaction cocktail as recommended by the manufacturer and incubated for 30 min in the dark at 20°C. After three PBS washes, the cells were counterstained with Hoechst 33342 (Invitrogen, Waltham, MA, USA). The cells were then put on glass slides, mounted in PBS, and examined with an LSM 700 laser‐scanning confocal microscope using a C‐Apochromat 10× objective (Zeiss, Jena, Germany). The average fluorescence signal intensity was calculated after images were obtained with the same laser intensity. Images were analyzed using ZEN 2012 Blue (Zeiss, Jena, Germany) and ImageJ software 1.53e (National Institutes of Health, Bethesda, MD, USA).

### 2.12 Real‐time RT‐PCR analysis of mRNA levels

Following the manufacturer's instructions, total RNA was extracted from cells using the QIAzol lysis reagent (79306, Qiagen, Hilden, Germany), and it was stored at −70°C until use. Following the manufacturer's instructions, cDNA was produced from total RNA (2 μg) using TOPscript™RT Drymix (RT200, Enzynomics, Daejeon, Korea). RT‐PCR in real time was carried out using the QuantiSpeed SYBR NO‐ROX kit (QS105‐10, Phile Korea, Seoul, Korea). For the purpose of normalizing the data, endogenous glyceraldehyde 3‐phosphate dehydrogenase (*GAPDH*) (accession number: NM 001256799) was added. The target gene mRNA levels were normalized to those seen in controls. The following were the primer sequences: Bax (accession number: NM 138763) Forward: CCCGAGAGGTCTTTTTCCGAG, Reverse: CCAGCCCATGATGGTTCTGAT.

### 2.13 Flow cytometric analysis

HaCaT cells were cultured in 60 mm dishes and treated for 3 days (24 h pretreatment, followed by 2 days of blue light irradiation). After collecting the suspended medium, the cells were washed with 1 mL of PBS and collected in 15‐mL tubes. The tubes were then centrifuged for 3 min at 212*g*. Trypsin (500 μL) was used to harvest cells, and the supernatant was collected after 3 min of centrifugation at 212*g*. The cell pellets were then resuspended in 200 μL of 1× annexin binding buffer containing double‐distilled water treated with diethyl pyrocarbonate (DEPC‐DW; C‐9030, Bioneer, Daejeon, Korea). Dead Cell Apoptosis Kit with Annexin V FITC and PI for flow cytometry (V13242; Invitrogen, Waltham, MA, USA) was used to stain the cells, during which the cells were incubated for 15 min at 20°C in the dark. The samples were kept on ice after gentle mixing with 400 μL of 1× annexin binding buffer. At least 30,000 stained cells were counted and subjected to flow cytometric analysis (CytoFLEX; Beckman Coulter, Brea, CA, USA).

### 2.14 Statistical analysis

The data are described as the standard error of the mean. The Student's *t* test was used to examine differences between the two groups. One‐way analysis of variance was used to compare various groups, followed by Tukey's multiple comparison test, both of which were performed using GraphPad Prism (5.0) (GraphPad, La Jolla, CA, USA). Statistical significance was defined at a *p*‐value less than 0.05.

## 3 RESULTS

### 3.1 Melatonin recovers cell viability and reduces ROS production induced by blue light irradiation

**FIGURE 1:**
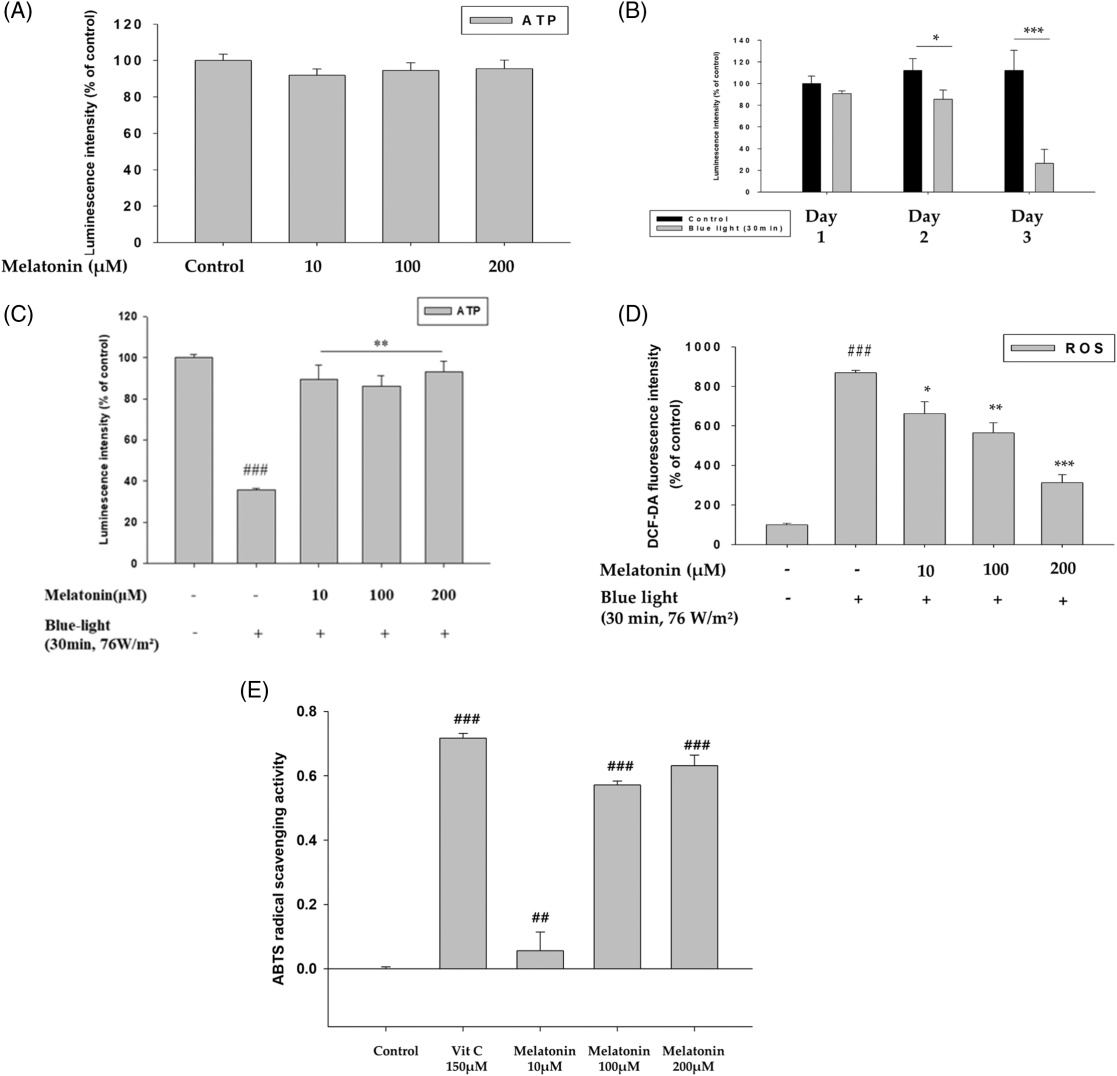
Effect of melatonin on the reduced cell proliferation and elevated ROS production induced by blue light. (A) HaCaT cells were pretreated with 10, 100, and 200 μM melatonin for 3 days. CellTiterGlo® was used for determining cell viability. (B) HaCaT cells were irradiated with photoreactor blue light (30 min, 76 W/m^2^) for 1, 2, and 3 days repeatedly, and the cell viability was measured using CellTiterGlo®. (C) Effects of melatonin on viability of HaCaT cells irradiated with blue light (30 min, 76 W/m^2^) were determined using CellTiterGlo®. (D) HaCaT cells were treated with melatonin at different concentrations. After 24 h, they were stained with DC‐FDA and irradiated with LED‐BL for 30 min, and fluorescent images were recorded. (E) Scavenging effects of melatonin on ABTS free radicals, 150 μM Vit C was used as a positive control. (##*p* < 0.01: vs. control, ###*p* < 0.001: vs. control, *p < 0.05: vs. blue light, **p < 0.01: vs. blue light, ***p < 0.001: vs. blue light).

Human keratinocytes (HaCaT) were treated with 10, 100, or 200 μM melatonin to determine cytotoxicity (Figure 1A). The viability of HaCaT cells did not decrease with melatonin treatment, indicating that melatonin is not cytotoxic to human keratinocytes (Figure 1A). To determine whether LED‐BL irradiation induces cytotoxicity and to determine the working doses of LED‐BL, HaCaT cultures were exposed to LED‐BL for different numbers of days (Figure 1B). Unlike the cells irradiated for 1 day, cell viability was significantly reduced in the groups irradiated with blue light for 2 and 3 days compared with the unexposed cells (Figure 1B). Exposure to LED‐BL for two repeated days (27.36 J/cm^2^) significantly reduced the viability of HaCaT cells (Figure 1B). Therefore, to test the protective effect of melatonin at different concentrations, 27.36 J/cm^2^ of energy was used for subsequent experiments. The viability of keratinocytes treated with LED‐BL at 27.36 J/cm^2^ and melatonin at different concentrations was measured relative to the control, which was treated only with LED‐BL (Figure 1C). Blue light‐induced cell damage is associated with ROS production.^9^ Therefore, we examined ROS production induced by LED‐BL and how it is affected by melatonin. Unlike in unexposed control cells, DCF‐DA fluorescence increased significantly when the cells were exposed to LED‐BL, indicating enhanced ROS generation (Figure 1D). In addition, melatonin treatment significantly reduced ROS production in a dose‐dependent manner (Figure 1D). We further performed 2,2′‐azino‐bis(3‐ethylbenzothiazoline‐6‐sulfonic acid) (ABTS) assay to examine whether melatonin possesses radical scavenging activity. Melatonin reduced ABTS cation radicals in dose‐dependent manner (Figure 1E). Melatonin in 200 μM concentration showed a similar level of radical scavenging activity to vitamin C, which is used as a positive control (Figure 1E). As can be seen from Figure 1, melatonin treatment helped human keratinocytes recover their viability by reducing the ROS production induced by LED‐BL.

### 3.2 Melatonin attenuates TRPV1–Hippo signaling induced by blue light

**FIGURE 2:**
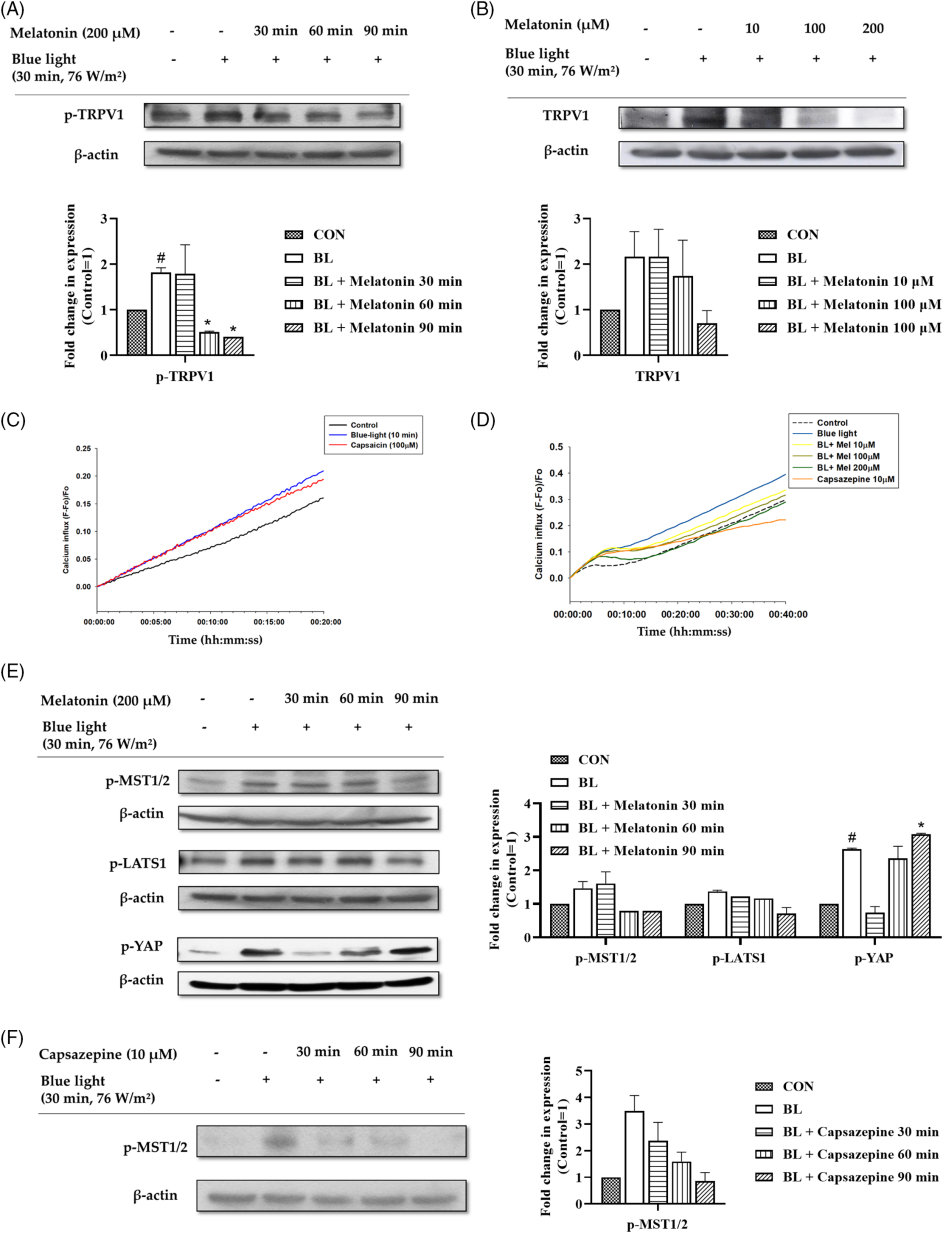
Effects of melatonin on blue light‐induced TRPV1–Hippo signaling. (A) HaCaT cells were irradiated with LED‐BL (76 W/m^2^) and treated with melatonin (200 μM) for 30, 60, and 90 min. The phosphorylated form of TRPV1 was analyzed using western blotting. (B) Cells were treated with melatonin and LED‐BL (76 W/m^2^) as indicated in the method. The cells were harvested immediately after incubation, and the protein levels of TRPV1 were detected using western blotting analysis. β‐Actin was used as a loading control. (C,D) Calcium influx into HaCaT cells was measured using the Fluo‐4 NW assay. After one hour of treatment with melatonin or capsazepine, the cells were washed with assay buffer and stained with Fluo‐4 reagent. Then, the cells were irradiated with blue light and treated again with melatonin or capsazepine. After treatment, the fluorescence intensities were measured immediately. (E) HaCaT cells were irradiated with LED‐BL (76 W/m^2^) and treated with melatonin (200 μM) for the indicated time duration. The phosphorylated form of Hippo signaling molecules were then analyzed using western blotting. (F) Cells were irradiated by LED‐BL (76 W/m^2^) and treated with capsazepine (10 μM) for 30, 60, and 90 min. The levels of p‐MST1/2 were then measured using western blotting. (E, F) β‐Actin was introduced as the loading control.

LED‐BL has been reported to induce ROS production through TRPV1 signaling, resulting in a decrease in cell proliferation.^9^ In this study, we verified that melatonin inhibits ROS production, thereby protecting against cell damage induced by LED‐BL. Therefore, we assumed that melatonin improved the viability of keratinocytes exposed to LED‐BL by inhibiting TRPV1 activation. Specifically, we found that the phosphorylation level of TRPV1 increased upon LED‐BL irradiation and decreased after melatonin treatment in a time‐dependent manner (Figure 2A). The expression of TRPV1 also increased upon LED‐BL irradiation and decreased following melatonin treatment in a dose‐dependent manner (Figure 2B). Next, we analyzed LED‐BL‐induced calcium influx by using capsaicin, an agonist of TRPV1, as a positive control. Notably, the levels of calcium influx in irradiated cells were higher than those in cells treated with capsaicin (Figure 2C). We then compared the calcium influx in LED‐BL‐exposed control cells with that in melatonin‐treated cells exposed to LED‐BL, where capsazepine, an antagonist of TRPV1, was used as the negative control. Both capsazepine‐ and melatonin‐treated HaCaT cells exhibited decreased calcium influx (Figure 2D).

MST1/2 play a critical role in Hippo signaling and are important in controlling cell proliferation and growth. MST1/2 can be activated through various stimuli, including ROS and Ca^2+^.^13^ In this study, phosphorylation of MST1/2, LATS1, and YAP increased after LED‐BL irradiation and decreased after melatonin treatment. However, YAP phosphorylation recovered after 90 min of melatonin treatment. This may be attributed to other mechanisms independent of the core kinase cascade (Figure 2E). To date, there have been no reports relating TRPV1 to MST1/2 signaling in human keratinocytes. Thus, we examined the involvement of TRPV1 in MST1/2 activation. To this end, we treated HaCaT cells with capsazepine and measured the phosphorylation levels of MST1/2 (Figure 2F). LED‐BL irradiation increased the phosphorylation levels of MST1/2, whereas capsazepine treatment decreased the phosphorylation level as treatment time increased (Figure 2F). Collectively, these results suggest that TRPV1 activation by LED‐BL induces the activation of MST1/2 and its downstream molecules, whereas melatonin attenuates these effects.

### 3.3 Melatonin reduces YAP–p73 interaction induced by long‐term exposure to blue light

**FIGURE 3:**
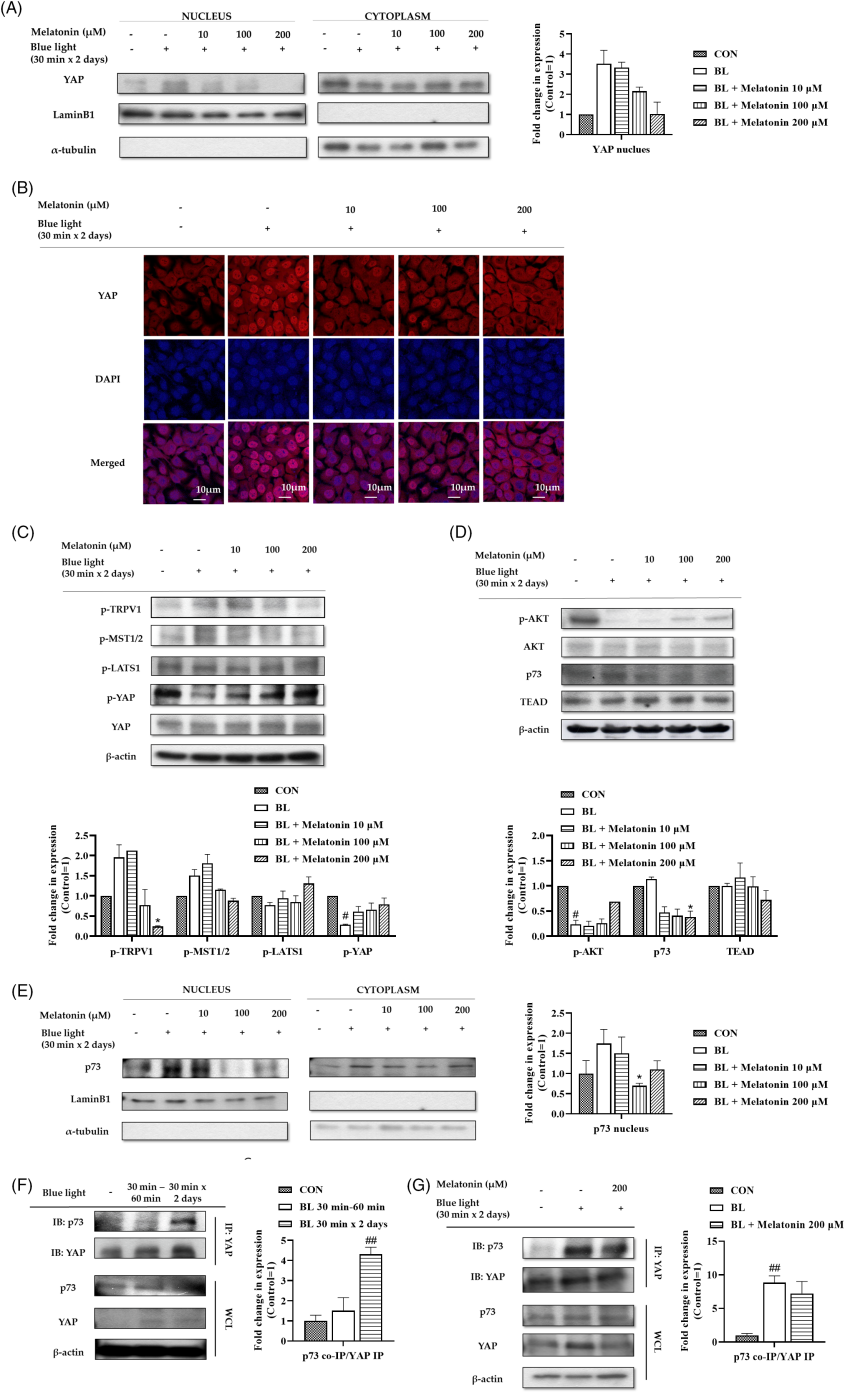
Effect of melatonin on cells subjected to long‐term exposure of blue light. (A) Cells were irradiated with blue light for long periods and treated with melatonin at the indicated concentrations. After the treatment, nuclear and cytoplasmic YAP was analyzed using western blotting. Lamin B1 and α‐tubulin were used as loading controls for the nucleus and cytoplasm, respectively. (B) The fluorescence intensity of YAP and DAPI were measured in response to both blue light irradiation and melatonin treatment using immunofluorescence assay. (C,D) Cells were treated with melatonin and irradiated with blue light as indicated in the method. The cells were harvested immediately after incubation, and the protein levels were detected using western blotting analysis. β‐Actin was introduced as a loading control. (E) Nuclear and cytoplasmic p73 was analyzed using western blotting. Lamin B1 and α‐tubulin were introduced as loading controls for the nucleus and cyto‐plasm, respectively. (F, G) Using whole‐cell lysates, co‐immunoprecipitation was performed to analyze the interaction between YAP and p73. The samples were immunoprecipitated with anti‐YAP antibody, and western blot was probed with anti‐p73 and anti‐YAP antibodies.

We demonstrated that LED‐BL irradiation induced YAP phosphorylation, whereas melatonin reduced its effect. The nuclear and cytoplasmic distribution of YAP is regulated by phosphorylation at specific locations, which control YAP stability and transcriptional activation.^16^, ^29^, ^30^ Therefore, we determined the effects of LED‐BL and melatonin on the cellular localization of endogenous YAP within HaCaT cells. As we determined that irradiation with LED‐BL induced YAP phosphorylation (Figure 2), we expected that it would also be sufficient to reduce YAP nuclear translocation by sequestering YAP in the cytoplasm. However, the nuclear translocation of YAP was increased by long‐term LED‐BL irradiation, whereas melatonin treatment with LED‐BL irradiation reduced its translocation (Figure 3A). Similar results were obtained using immunofluorescence assay (Figure 3B). To investigate the mechanism of melatonin action following long periods of LED‐BL irradiation, we examined the effects of LED‐BL and melatonin on the TRPV1–Hippo signaling pathway. Long periods of LED‐BL irradiation induced the phosphorylation of TRPV1 and MST1/2, whereas melatonin treatment reduced their activation (Figure 3C). Notably, there was no change in the phosphorylation levels of LATS1 after either LED‐BL irradiation or irradiation combined with melatonin treatment. Although YAP phosphorylation was reduced by LED‐BL irradiation, melatonin treatment increased its phosphorylation (Figure 3C). It is known that DNA damage induces YAP–p73 interaction, leading to cell apoptosis; nonetheless, active Akt can attenuate this action by promoting YAP cytoplasmic localization through phosphorylation on S127.^22^ MST1/2 activation inhibits Akt activation, which in turn reduces cell proliferation.^31^, ^32^ Therefore, we hypothesized that LED‐BL irradiation inhibits Akt phosphorylation. As expected, LED‐BL irradiation inhibited Akt activation, whereas its activation recovered with melatonin treatment (Figure 3D). In addition, there was no change in the expression of TEAD after LED‐BL irradiation and melatonin treatment, although p73 expression increased upon LED‐BL irradiation and decreased upon melatonin treatment (Figure 3D). We next identified the subcellular location of p73. p73 mainly located in the nucleus after long‐term exposure to LED‐BL (Figure 3E). However, after the treatment of melatonin with LED‐BL, nuclear translocation of p73 decreased (Figure 3E). To directly determine the effect of LED‐BL over longer periods of exposure, we irradiated cells with LED‐BL for short (30 min) or long periods (30 min, two repeated times). Co‐immunoprecipitation (Co‐IP) was then performed to detect the interaction between YAP and p73. As shown in Figure 3F, we found that the YAP–p73 complex formation increased after long‐term exposure to LED‐BL, whereas short‐term exposure to LED‐BL caused no significant difference compared to that observed in the control group (Figure 3F). Conversely, melatonin treatment significantly attenuated the interaction between YAP and p73 (Figure 3G). Figure 3 shows that long‐term exposure to LED‐BL induces YAP–p73 interaction, whereas melatonin treatment reduces this effect by inducing Akt activation.

### 3.4 Short‐term exposure of blue light reduces YAP–TEAD interaction by inhibiting YAP nuclear translocation

**FIGURE 4:**
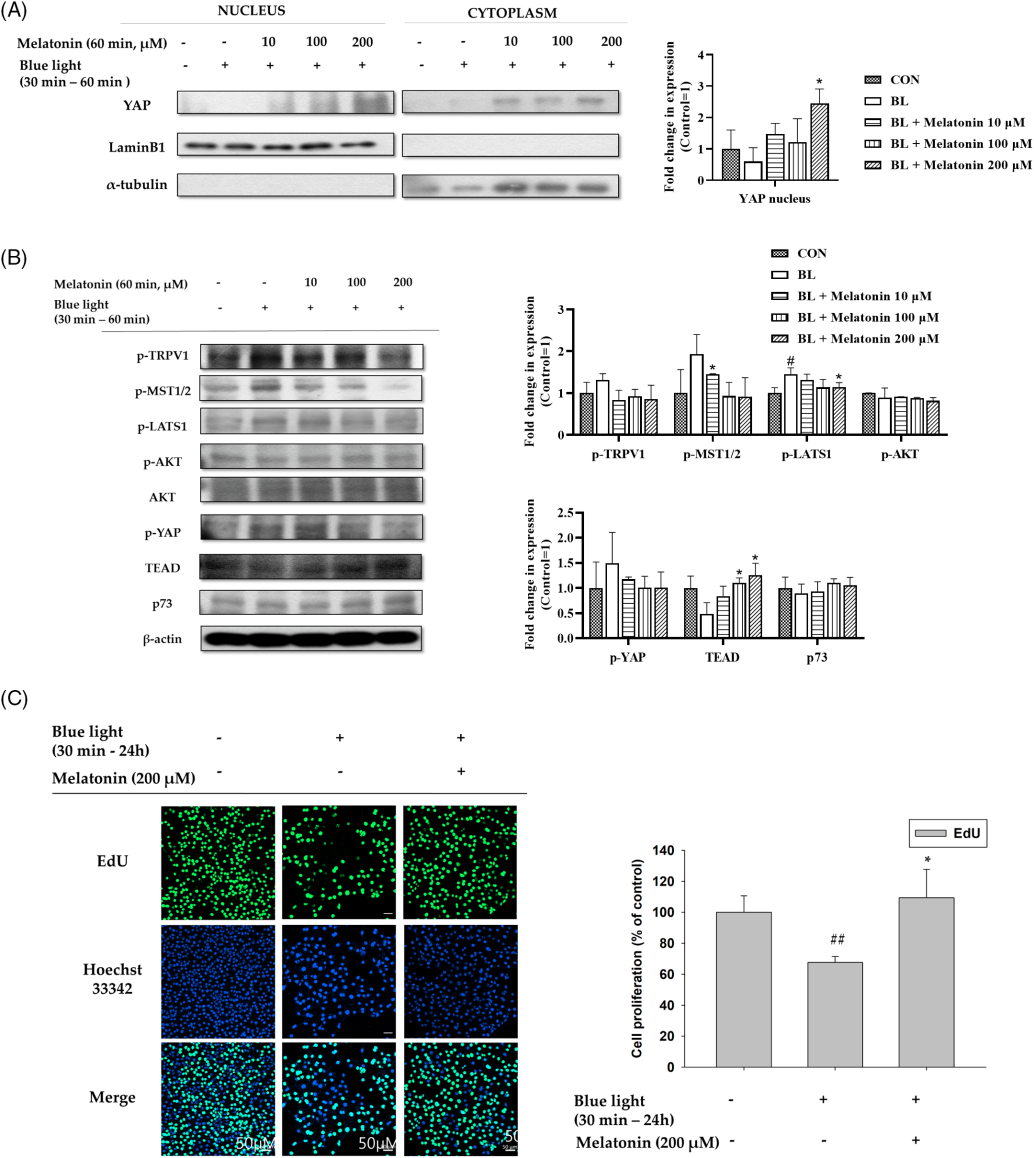
Effect of melatonin treatment on cells irradiated with blue light for a short term. (A) Cells were irradiated with blue light for 30 min and then treated with melatonin at the indicated concentrations for 60 min. Western blot evaluation of YAP nuclear translocation was performed after the treatment. Lamin B1 and α‐tubulin were used as loading controls for the nucleus and cytoplasm, respectively. (B) The cells were irradiated with LED‐BL for 30 min and then treated with melatonin for 60 min. After the incubation, the cells were harvested, and western blotting analysis was used to determine the protein levels. β‐Actin was introduced as a loading control. (C) HaCaT cells were irradiated with LED‐BL and then treated with melatonin at the indicated concentrations for 24 h. Cell proliferation was detected using an EdU assay. (##*p* < 0.01: vs. control, *p < 0.05: vs. blue light).

We first assumed that LED‐BL exposure inhibits YAP–TEAD interaction by blocking YAP nuclear translocation. However, we determined that long‐term exposure to LED‐BL instead induces YAP nuclear translocation, thus promoting YAP–p73 interaction (Figure 3). Therefore, we next examined the mechanism of action of YAP following short‐term exposure to LED‐BL and elucidated how this activity is impacted by melatonin. A short period of LED‐BL irradiation of human keratinocytes inhibited YAP nuclear translocation, whereas melatonin treatment antagonized the blue light irradiation effect (Figure 4A). Next, we investigated the effect of short‐term blue light exposure on TRPV1–Hippo signaling. Human keratinocytes were irradiated with LED‐BL for 30 min and treated with melatonin for 1 h. Short‐term exposure to LED‐BL induced the phosphorylation of TRPV1, MST1/2, LATS1, and YAP, while melatonin treatment mitigated these actions (Figure 4B). Unlike long‐term exposure to LED‐BL, a short period of LED‐BL inhibited the expression of TEAD, whereas melatonin treatment increased its expression. There was no effect on Akt or p73 expression (Figure 4B). YAP–TEAD interaction promotes cell proliferation.^16^, ^33^ In this study, short‐term exposure to LED‐BL reduced HaCaT cell proliferation, whereas melatonin treatment rescued this effect (Figure 4C). These findings demonstrate that short‐term exposure to LED‐BL inhibits YAP–TEAD interaction, thereby reducing cell proliferation; nonetheless, melatonin attenuates these effects.

### 3.5 Long‐term exposure to blue light induces DNA damage in human keratinocytes

**FIGURE 5:**
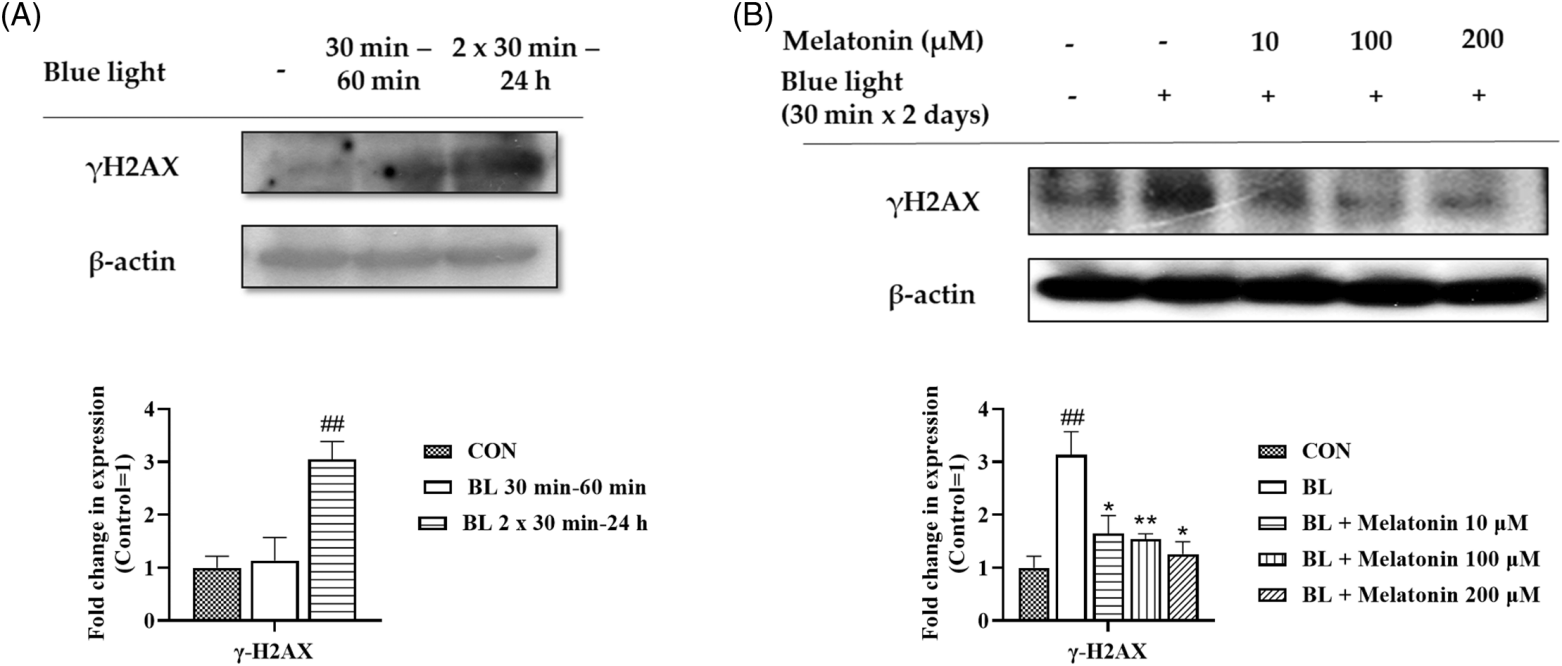
Effect of blue light irradiation on DNA damage. (A) Cells were irradiated with LED‐BL for the indicated time durations. The cells were harvested immediately after incubation, and western blotting analysis was performed to analyze the protein levels of γH2AX. (B) HaCaT cells were treated with melatonin and LED‐BL as indicated in the method. Expression of γH2AX was determined using western blot analysis. As the loading control, β‐actin was introduced.

We found that the mechanism of action of blue light in HaCaT cells depends on the duration of exposure. Long‐term exposure to LED‐BL induced YAP–p73 interaction, while melatonin treatment attenuated this effect by promoting Akt activation. We assumed that this could be attributed to DNA damage. Therefore, we determined the occurrence of DNA damage by examining the expression of γH2AX. Western blotting was used to detect the stimulation of γH2AX expression following LED‐BL irradiation. γH2AX expression increased in response to long‐term LED‐BL exposure, whereas short‐term irradiation with LED‐BL did not cause a significant effect compared to that observed in the control group (Figure 5A). Melatonin treatment following prolonged LED‐BL irradiation reduced γH2AX expression (Figure 5B). The results of this experiment demonstrated that long‐term exposure to LED‐BL irradiation induced DNA damage, and melatonin not only blocked the damage induced by YAP–p73 interaction, but also reduced DNA damage.

### 3.6 Melatonin reduces cell apoptosis by attenuating p73–YAP target gene

**FIGURE 6:**
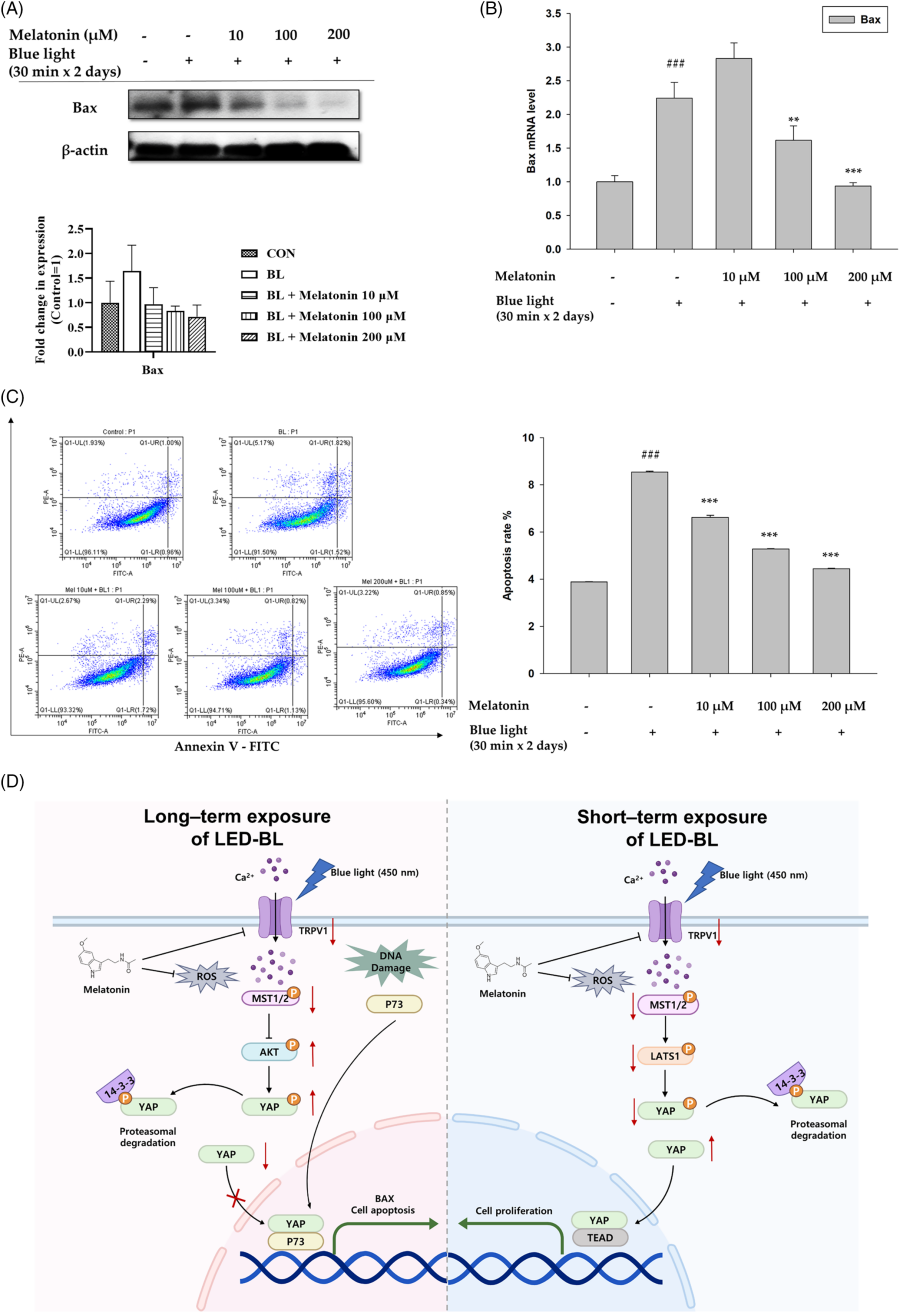
Effect of blue light irradiation on DNA damage. (A) Bax expression levels in HaCaT cells irradiated with LED‐BL, with or without melatonin treatment, were determined using western blot analysis. β‐Actin was used as the loading control. (B) mRNA levels of Bax were analyzed using qRT‐PCR. As the housekeeping gene, glyceraldehyde 3‐phosphate dehydrogenase (GAPDH) was used. (C) Effect of melatonin on LED‐BL‐induced cell apoptosis was assessed using flow cytometry. (###*p* < 0.011: vs. control, ***p < 0.001: vs. blue light). (D) Schematic diagram showing the action mechanism of melatonin in blue light‐irradiated human keratinocytes.

YAP accelerates cell apoptosis by interacting with p73, resulting in increased Bax (the target of p73) expression.^21^, ^34^ To detect Bax expression, HaCaT cells were treated with melatonin and irradiated with LED‐BL for the indicated time durations. Bax expression increased in LED‐BL‐irradiated untreated cells but decreased in LED‐BL‐irradiated cells treated with melatonin (Figure 6A). The mRNA levels of Bax were also measured, and similar results were obtained using quantitative PCR analysis (Figure 6B). A cell apoptosis assay was performed via flow cytometric analysis to directly observe the nature of cell apoptosis. During irradiation with LED‐BL, 8.31% of HaCaT cells underwent apoptosis. However, following melatonin treatment, the apoptotic rate decreased to 5.22% (Figure 6C). Taken together, melatonin inhibited the YAP–p73 proapoptotic pathway in LED‐BL‐irradiated human keratinocytes. Collectively, melatonin has the potential to protect against and/or mitigate skin damage induced by LED‐BL. In addition, as shown in Figure 6D, we summarized the possible mechanism of action of melatonin under various conditions.

## 4 DISCUSSION

Our skin is exposed to blue light from the digital screen and sunlight. Because blue light has a relatively short wavelength, it generates significant energy. Therefore, exposure to blue light may cause substantial long‐term harm to the skin.^9^, ^34^ Blue LED light, particularly, has been shown to inhibit the proliferation of human keratinocytes. It is known that blue light produces ROS through activating TRPV1 in human keratinocytes.^9^ Melatonin is known to be a potent antioxidant that reduces light‐induced damage during the day.^34^ Therefore, in this study, we investigated the role of melatonin in mitigating blue light‐induced damage to human keratinocytes.

Our study demonstrates that blue light decreases the viability of human keratinocytes and increases the production of ROS. Concurrently, melatonin treatment protected human keratinocytes from blue light by inhibiting ROS production, leading to recovery of cell viability. As we are exposed to blue light damage frequently throughout the day, these findings are critical for developing appropriate treatment strategies.

Previous studies have shown that TRPV1 is targeted by blue light. It has been linked to several aspects of skin biology, including skin cell survival, proliferation, apoptosis, proinflammatory responses, and pigmentation.^9^, ^31^, ^35^ Melatonin is known to block TRPV1 activation in dorsal root ganglion cells, thereby inhibiting neuronal death.^36^ Additionally, there is a report on melatonin directly interacting with Ile‐ and Asn‐residues in all TRPV1 chains, supporting the hypothesis that melatonin acts directly on TRPV1.^37^ However, thus far, there has been no research on the relationship between melatonin and TRPV1 receptors in human keratinocytes. In this study, we demonstrated that melatonin inhibited TRPV1 activation by suppressing ROS production. Notably, ROS produced through TRPV1 leads to the phosphorylation of more TRPV1 as part of the feedback process. Therefore, the reduction in cellular ROS by melatonin is essential to inhibiting TRPV1 activation. Further investigation, including protein binding assays or mutational studies, would be necessary to conclusively determine whether melatonin directly interacts with TRPV1 or exerts its effect through alternative receptor pathways.

An increase in calcium levels activates Hippo signaling, leading to a decrease in proliferation.^13^ However, as there is no systematic relationship between TRPV1 and MST1/2, which are the key mediators of Hippo signaling, we investigated whether MST1/2 activation could be mediated by the calcium influx induced by TRPV1 activation. Our findings show that blue light activates Hippo signaling mediators, whereas melatonin inhibits their actions. Furthermore, the phosphorylation levels of YAP recovered after 90 min of incubation with melatonin, which we attributed to other independent mechanisms in the core kinase cascade. In addition, we found that MST1/2 activation is reduced with capsazepine treatment, indicating that MST1/2 is activated by TRPV1 activation.

YAP induces cell proliferation when it is translocated to the nucleus, where it forms a complex with TEAD.^38^ We first predicted that blue light irradiation would reduce YAP nuclear translocation, blocking the interaction between YAP and TEAD. However, prolonged exposure to blue light induced the nuclear translocation of YAP. Therefore, we assumed that prolonged exposure to blue light has a different mechanism of action in human keratinocytes. Notably, under conditions of DNA damage, phosphorylation of YAP by c‐Abl kinase was shown to be necessary for changing YAP activity from anti‐apoptotic to pro‐apoptotic.^39^, ^40^ Additionally, DNA damage induces the interaction of YAP with p73, further promoting apoptosis.^41^, ^42^ We next characterized the interaction between YAP and p73 through Co‐IP. We found that the YAP–p73 complex induces the expression of Bax, an apoptotic gene. Melatonin treatment attenuated these effects by inhibiting TRPV1 activation and its downstream molecules, such as MST1/2. Specifically, MST1/2 activation inhibits Akt activation, leading to decreased cell proliferation.^29^, ^32^ Melatonin treatment recovered this Akt activity by reducing MST1/2 activation. These effects eventually inhibit YAP nuclear translocation, blocking its association with p73.

We further investigated the effects of short‐term blue light irradiation. Cells that underwent only short‐term exposure to blue light did not show significant DNA damage. Based on this result, we predicted that the mechanism of action would be different among cells receiving long‐term blue light exposure. Following short‐term exposure, nuclear translocation of YAP was reduced; however, melatonin rescued this effect. Furthermore, after 30 min of blue light irradiation, YAP expression decreased; nonetheless, melatonin treatment augmented YAP expression. Additionally, unlike cells subjected to long‐term exposure, short‐term irradiation with blue light induced LATS1 activity, leading to phosphorylation of YAP in the serine 127 region, although there was no difference in Akt activation and p73 expression. Unlike in long‐term exposed cells, phosphorylation of YAP in the serine 127 region was induced by LATS1 and not Akt. Therefore, we concluded that melatonin recovers proliferation by promoting YAP–TEAD interaction in cells irradiated for short periods.

In this study, we examined the specific mechanisms of action associated with different amounts of blue light exposure. In addition, we revealed the mechanism of action of melatonin under various conditions. However, it is possible that the observed effects were mediated directly by melatonin or indirectly through its metabolites, such as indolic or kynuric derivatives, which have been implicated in modulating oxidative stress and cellular responses. Given the rapid metabolism of melatonin in the skin and keratinocytes, the protective effects against blue light‐induced cell damage may be attributed to either melatonin itself or its downstream metabolites. Further studies are required to delineate the precise contribution of melatonin versus its metabolites.^43^ Blue light irradiation causes an imbalance in the distribution of calcium, which can disrupt normal skin homeostasis. Therefore, finding a molecule with anti‐blue light effects is important for protecting the skin in everyday life. We showed that melatonin exerts protective effects against oxidative stress as well as reduced cell viability. Thus, this study describes the beneficial effects of melatonin and its potential use as a treatment for skin cell damage induced by blue light irradiation.

## FUNDING INFORMATION

This research was supported by the Technology Development Program (S3430878), funded by the Ministry of SMEs and Startups (MSS), Republic of Korea; and a grant from the Basic Science Research Program through the National Research Foundation of Korea (NRF) funded by the Ministry of Science and Technology Information and Communication (grant no. RS‐2023‐00246887).

## CONFLICT OF INTEREST STATEMENT

The authors declare no conflicts of interest. The funders had no role in the study design, collection, analyses, or interpretation of data, writing of the manuscript, or decision to publish the results.

## Data Availability

The data that support the findings of this study are available from the corresponding author upon reasonable request.
